# Supplementary material for: A double dissociation between savings and long-term memory in motor learning
Source: PLoS Biol. 2023 Apr 27;21(4):e3001799. doi: 10.1371/journal.pbio.3001799 (PMC10138789; doi:10.1371/journal.pbio.3001799)
Supplement: S1 Fig — Here, we investigated whether the anti-savings found in Experiments 1/2 (most pronounced following an 800-trial washout) might be due to the prolonged 800-trial washout period strengthening the baseline, unadapted state to the point that it resists the formation of a temporally-persistent memory of adaptation during relearning. Previous literature suggests that this kind of repetition effect—a form of use-dependent learning [69,70]—tends to level off after only 50–150 trials [71]; thus, it should equally affect initial learning (which follows 220 baseline trials) and relearning after 800 washout trials, suggesting no net effect in the anti-savings we observe. However, this use-dependent learning effect has not been studied within the specific context of our task. Thus, in Experiment S1, we examined 12 new participants who adapted to a 30° visuomotor rotation following an 800-trial baseline period, to match the long washout period in Experiments 1 and 2. We found that the prolonged baseline in Experiment S1 (light gray) did not reduce the temporally-persistent component during adaptation compared to the shorter, 220-trial baseline in Experiments 1 and 2 (dark gray); instead, relearning after an 800-trial washout in Experiments 1 and 2 led to significant reductions in temporally-persistent adaptation, as we discuss in the main text. Together, these findings show that anti-savings in temporally-persistent adaptation were not due to the use-dependent learning during the long 800-trial washout period. (a) Comparison of average adaptation curves for (i) initial learning after 800 baseline trials from Experiment S1 (light gray), (ii) initial learning after 220 baseline trials from Experiments 1/2 (dark gray), and (iii) relearning after 800 washout trials from Experiments 1/2 (blue). (b) Close-up of the adaptation phase, with temporally-persistent measurements indicated by the empty circles as in Fig 3A. Note the similarity between the adaptation curves for the initial lear [file pbio.3001799.s001.pdf]

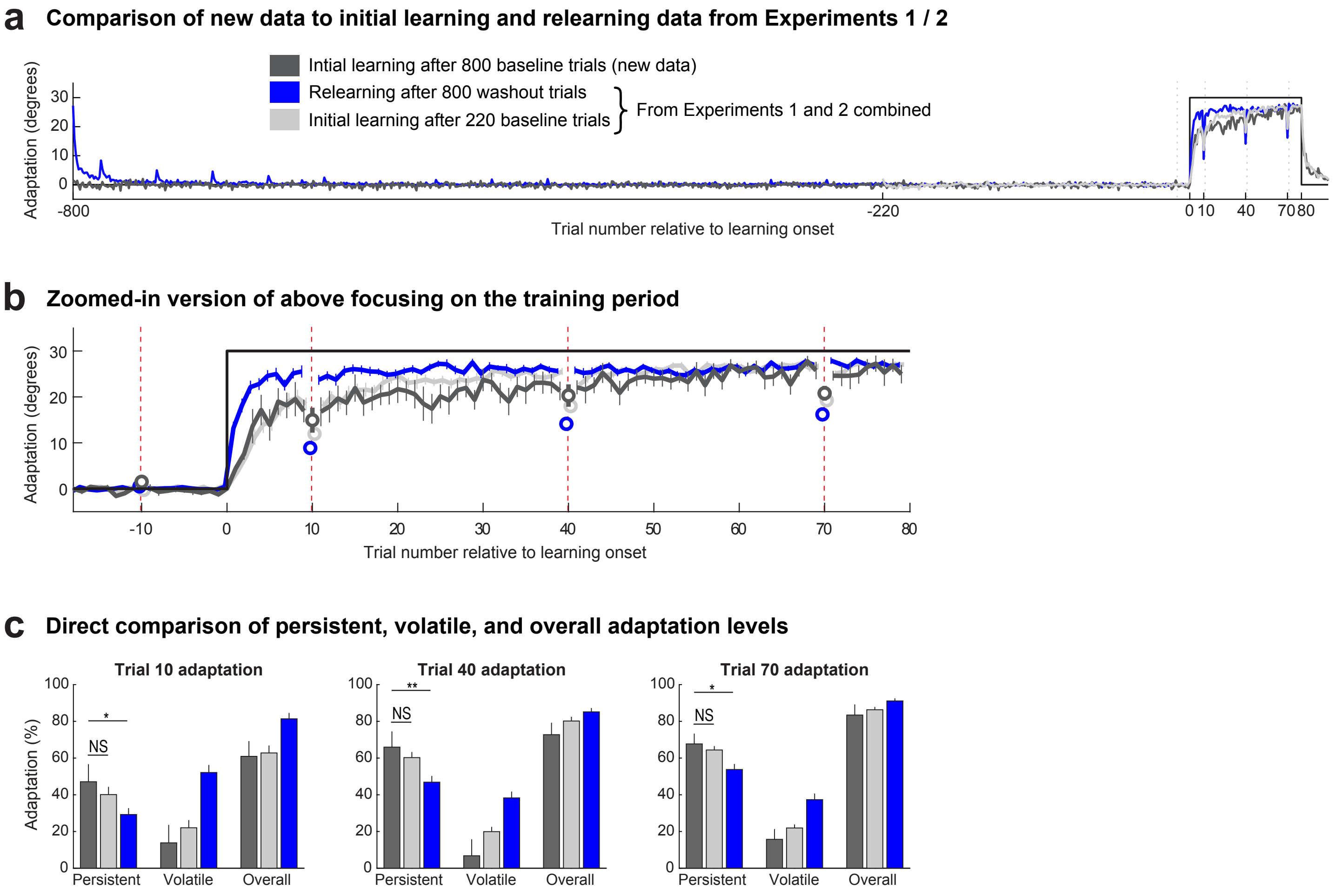

S1 Fig. *Anti-savings in temporally-persistent adaptation cannot be explained by prolonged reaching under baseline conditions.* Here we investigated whether the anti-savings found in Experiments 1 / 2 (most pronounced following an 800-trial washout) might be due to the prolonged 800-trial washout period strengthening the baseline, unadapted state to the point that it resists the formation of a temporally-persistent memory of adaptation during relearning. Previous literature suggests that this kind of repetition effect – a form of use-dependent learning (Classen et al., 1998; Diedrichsen et al., 2010) – tends to level off after only 50-150 trials (Verstynen & Sabes, 2011); thus, it should equally affect initial learning (which follows 220 baseline trials) and relearning after 800 washout trials, suggesting no net effect in the anti-savings we observe. However, this use-dependent learning effect has not been studied within the specific context of our task. Thus, in Experiment S1, we examined 12 new participants who adapted to a 30° visuomotor rotation following an 800-trial baseline period, to match the long washout period in Experiments 1 and 2. We found that the prolonged baseline in Experiment S1 (light gray) did not reduce the temporally-persistent component during adaptation compared to the shorter, 220-trial baseline in Experiments 1 and 2 (dark gray); instead, relearning after a 800-trial washout in Experiments 1 and 2 led to significant reductions in temporally-persistent adaptation, as we discuss in the main text. Together, these findings show that anti-savings in temporally-persistent adaptation were not due to the use-dependent learning during the long 800-trial washout period.

(a) Comparison of average adaptation curves for (i) initial learning after 800 baseline trials from Experiment S1 (light gray), (ii) initial learning after 220 baseline trials from Experiments 1 / 2 (dark gray), and (iii) relearning after 800 washout trials from Experiments 1 / 2 (blue).

(b) Close-up of the adaptation phase, with temporally-persistent measurements indicated by the empty circles as in Figure 3A. Note the similarity in the adaptation curves for the both initial learning cases (both after 220 and 800 trials of baseline) in contrast to the relearning curve. Errorbars indicate SEM; red lines indicate 60-second delays used to isolate temporally-persistent adaptation.

(c) Comparison of the levels of overall, temporally-persistent, and temporally-volatile adaptation for these three cases, at trials 10, 40, and 70 after the onset of the visuomotor rotation perturbation. Temporally-persistent adaptation displays no signs of reduction after the 800-trial baseline (new data) relative to the 220-trial one (Exp. 1/2 data); however, it is significantly higher than temporally-persistent adaptation during relearning after the 800-trial washout in Exp. 1/2. * $p<0.05$; ** $p<0.01$. Underlying data supporting this figure can be found in files Exp_1_2_data.mat and Exp_S1_data.mat.
