# Supplementary material for: A double dissociation between savings and long-term memory in motor learning
Source: PLoS Biol. 2023 Apr 27;21(4):e3001799. doi: 10.1371/journal.pbio.3001799 (PMC10138789; doi:10.1371/journal.pbio.3001799)
Supplement: Attachment — Submitted filename: response_to_reviewers.pdf [file pbio.3001799.s002.pdf]

Dear Editor,

We would like to thank the reviewers for their highly constructive feedback on our manuscript “A Double Dissociation between Savings and Long-Term Memory in Motor Learning” and for the positive tone of their reviews. For example, Reviewer 1 mentioned that “The phenomenon itself is intriguing, and the result will point to a novel mechanism of how the motor memory is formed”; Reviewer 2 said that “This study is interesting and timely … The 60 sec break manipulation is very elegant”, and Reviewer 3 stated that “The study is novel and certainly relevant to the field”. The reviewers also pointed out a few issues, including possible alternative explanations, methods clarifications, and better placement of our findings in the wider context of the learning and memory literature outside of motor learning, all of which we have fully addressed in this letter. As a result, the manuscript is greatly improved. For convenience, we outline our responses to the main issues that were raised below, before addressing all of the Reviewer comments in detail.

Reviewers 1 and 2 suggested that the anti-savings effect in temporally-persistent adaptation that we observed after the 800-trial washout might be explained by the prolonged duration of this washout period in which the same action – reaching for the target on no-rotation trials – was repeated, creating a use-dependent bias. This is a possibility as repeated reaches have been shown to bias reaching direction (Diedrichsen et al., 2010; Verstynen & Sabes, 2011). However, we note that in our experiments, the initial learning to which relearning is compared also follows a prolonged no-rotation period, and previous research in reaching suggests that use-dependent effects plateau before the 220-trial duration of that period. Thus any use-dependent biases would be expected to affect initial learning and 800-trial relearning to the same extent and therefore not contribute to the anti-savings effect that we observe. However, because the effect of long-term use-dependent biases has not been previously investigated for periods as long as 800 trials, we carried out a new experiment which examined initial learning after a baseline period matching the duration of the 800-trial the long washout period, to check whether, as predicted, going from 220 to 800 trials has little effect in slowing initial learning. The results of this new experiment showed that the anti-savings effect originally observed was preserved when referenced to the new 800-trial initial learning condition and that the levels of initial adaptation following this alternate 800-trial baseline matched that observed after 220 baseline trials.

Reviewers 1 and 3 asked for better explanation of the differences in the design of experiment 4, where we measured long-term retention, compared to experiments 1-3, where we measured savings, and we have now provided this, both in the response letter and in the Results and Methods sections of the revised manuscript.

The reviewers also had a series of comments placing our findings within the existing literature, which we have now provided. For example, Reviewer 1 asked whether the increased learning rate for temporally-volatile adaptation should itself be seen as a form of long-term memory; we discuss how this could indeed be considered long-term memory but would be clearly distinct from the prevailing understanding of a consolidated memory robust to interference. Reviewer 2 astutely pointed out a parallel between the fast-learning/volatile vs. slow-learning/persistent dissection that we examine and previous work on contextual interference. We now discuss the possibility of a link in the Discussion, delineating how the dissection of memory into volatile vs. persistent components may explain previously reported contextual interference effects. Reviewer 3 asked us to better frame and provide more information up front on long-term memory as well as the memory dissection that we perform, and the abstract and introduction are now improved as a result.

We believe these comments – and our response to them, including a new experiment to assess use-dependent effects on the 800-trial relearning condition, clarifications of our experiment design, better discussion of specific findings, and improved placing of our work within the wider literature – have resulted in a greatly improved manuscript. We truly appreciate this help. We address reviewers’ specific comments point-by-point on the subsequent pages of this letter. For readability, we display reviewer comments in blue and our responses in black.

Sincerely,

Alkis M. Hadjiosif, J. Ryan Morehead, and Maurice A. Smith

Reviewer #1: Speed of learning is faster for the tasks which has been learned before, known as savings. In motor learning which of the multiple motor learning systems is the main contributor to the savings is still under debate. In this manuscript, authors claim that the temporally volatile component of motor memory contribute to the savings, but the temporally persistent memory rather disrupts the savings.

Participants learned a straight reaching under 30deg visuomotor rotation. After the learning, participants underwent a washout phase. A one-minute break was occasionally inserted during the washout phase, and the aftereffect measured immediately after this mini-break was defined as the component from the temporally persistent memory, meaning that it is robust to temporal perturbation.

Following are the main findings.

1) Time constant between temporally persistent and volatile motor memories indeed had different time constants.

2) Even after 800 trials of washout, which eliminated the persistent component of memory (or at least brought the memory back to zero), the savings was still observed. Temporally persistent memory did not show savings.

3) Such savings existed when the task was designed to exclude any explicit component (i.e. "move your hand to the target" instruction), suggesting that the savings is not due to the explicit strategy.

4) Temporally persistent memory explains the long-term retention of the motor memory, but the temporally volatile memory did not. Conversely, temporally persistent memory did not explain the savings, but the volatile memory does.

The authors conclude that the savings is not a manifesto of consolidated memory, but somehow arise from the increased learning rate when encountering the previously exposed perturbation pattern. The phenomenon itself is intriguing, and the result will point to a novel mechanism of how the motor memory is formed. The experiment is designed and carried out appropriately.

This is a nice summary of our manuscript. We thank the reviewer for the kind words here.

However, I feel there are couple of points to be clarified before recommending for publication, especially about the interpretation of the data.

Major

1.1 The finding of this paper is that, there is an unknown mechanism which heightens the learning rate of the temporally volatile learning system during re-learning of the perturbation, but does not for (what they call) temporally persistent learning system.

There must be some kind of long-term memory/storage mechanism which enables the savings to happen, but there is no guarantee that such long-term memory mechanism is captured by measuring the temporally persistent learning.

Therefore, lack of signature of savings in the temporally persistent learning measure does not necessary means that the memory in the initial learning has not entered the long-term memory system and has not been consolidated. Thus, claiming that savings does not require consolidation of memory is a bit far stretched.

To claim this, authors must show that the persistent memory measure indeed captures the sole consolidation mechanism for motor memory. Please clarify this point.

Here the reviewer asks us to clarify and defend the claim we make that savings does not require memory consolidation.

First, we fully agree with the reviewer that our results certainly cannot prove that memory consolidation makes no contribution to savings. Indeed, proving a negative like that would be essentially impossible, as the reviewer alludes to. We certainly do not intend for any reader to get the wrong idea about this, and thus in light of the reviewer’s criticism we have clarified this point in the text, as in shown revision excerpt(s) quoted below.

Discussion (lines 528-538):

"While the ability to establish this learning rate increase could be considered a type of long-term memory as it is long-lasting and must rely on a plastic change driven by experience, this ability is very different from the usual conception of a consolidated motor memory in which the trained actions themselves are remembered. Thus, our findings are consistent with a model in which implicit savings is based on an ability to improve the adaptation of actions, rather than a memory for the previous actions themselves. It is important to point out, however, that our findings do not show that consolidated memories are not formed or eliminate the possibility that they make some contribution to savings. For example, it is possible that explicit savings is driven by consolidated explicit memory. Even in our implicit adaptation data for which temporally-persistent relearning displays anti-savings, we cannot rule out the possibility that a consolidated temporally-persistent memory subcomponent exists and contributes to savings but that the effect is overwhelmed by a larger anti-savings effect from a different subcomponent. Instead, our findings demonstrate that consolidated memories are not required for savings."

While our results cannot prove that memory consolidation makes no contribution to savings, we maintain that they do demonstrate that memory consolidation is not *required* for savings. This is because savings is driven by temporally-volatile learning that as it is defined by the fact that it decays away in 60 seconds or less and thus cannot be considered as consolidated memory, which is universally defined as a "stable, long-lasting form" as opposed to a "temporary, labile memory" (Squire et al., 2015). This definition is in line with the fact that the word "consolidated" here literally means "with solidification". The reviewer, however, makes the point that in our experiments (and for savings in general) "There must be some kind of long-term memory/storage mechanism which enables the savings to happen." What the reviewer is astutely pointing out here is that the mechanism that promotes faster temporally-volatile learning must have a time course greater than that of the temporally-volatile learning itself. This is because this mechanism for savings must span the entire washout period between learning and relearning (which is clearly longer than the 60-second period over which temporally-volatile learning decays away, which is about 2 minutes for the 40-trial washout and about 40-45 minutes for the 800-trial washout in our study). However, this mechanism certainly cannot fit into the definition of consolidated memory, as it is a mechanism for increasing the learning rate of unconsolidated, temporally-volatile adaptation. As noted, this mechanism must span the entire washout period, and thus could be considered as a medium-term or long-term "memory" of sorts, but it is clearly distinct from the current conception of stable, consolidated memory in two ways: First, it is a mechanism for increasing the rate of change for what has been learned rather than promoting its stability. And second, it is a mechanism that involves motor memory that remains unstable and unconsolidated even during relearning.

1.2 In Experiment 4, authors show that the temporally persistent memory indeed predicts the retention of the memory 24 hrs later. However, the way the persistent memory is characterized here is completely different to Exp 1-3. In Experiment 1-3, persistent memory was measured during the course of washout, thus represents the memory that is robust to the recent washout experience. However, in Experiment 4, it is not measured during the washout, thus, it does not represent the robustness against the washout experience. Also, the timing from the previous learning experience is not specified, and 19 other directions are tested together without visual feedback. Thus, experiment 4 is too different to the other experiments by design, so the data from this experiment is difficult to be used to validate the result of the Experiment 1-3 or even to be discussed together with them. Since result of this experiment is used as important evidence to claim for the double dissociation, I advise the authors to retest this with the same setup as in Experiment 1-3.

We think that there is some confusion here which we will clarify below. It is critical to note that savings refers to faster learning **after** washout, whereas long-term retention refers to recall **without** washout. Thus, in the savings experiments (Expts 1-3) we measured temporally-persistent and temporally-volatile learning **after** washout, and in the long-term retention experiment (Expt 4) we measured temporally-persistent and temporally-volatile learning after training **without** washout. Had we measured temporally-persistent learning after washout in Expt 4 as the reviewer suggests above (note here that reviewer's comment above says "during washout" but we're

almost certain that he or she meant "after washout"), we would not have been able to examine long-term (24-hour) retention in Experiment 4, as the presence of washout precludes measurement of long-term retention.

Finally, we agree with the Reviewer that timing information is essential in characterizing these measurements as temporally-persistent in line with the definition used in Experiments 1-2 (i.e. using wait periods that were close to 1 minute from previous learning). We had already included this information in our initial submission's methods section: average break duration was 124±9s. Given a time constant for temporal decay of τ = 15-25s (Hadjiosif et al., 2012; Sing et al., 2009; Zhou et al., 2017), this would amount to ~6τ, allowing >99% of temporally-volatile adaptation decay, isolating temporally-persistent adaptation; this would be very similar to the effect of the 60s (~3τ) breaks, which would allow ~95% of temporally-volatile adaptation decay. Given the Reviewer's comment, and the importance of this information in characterizing these measurements are temporally-persistent adaptation, we have now included this information more prominently in the Results (lines 129-136) and in Fig. 5.

1.3 Authors showed that the temporally persistent component works in the opposite direction to savings during re-learning. However, the long washout period may have just simply made the persistent memory for the "baseline" and contributed to work in the direction of baseline, which is opposite to the direction of perturbation, during the re-learning. This is just like the persistent memory observed during the washout phase working in the direction of perturbation before being completely washed out. If this is the case, it will be difficult to say that the persistent component did not exhibit savings. Please discuss about this possibility. Perhaps the simple test would be to show how the persistent component is modulated during the initial learning, when preceded with baseline with different number of trials.

This is a conceptually sound criticism. Note that this point is very closely aligned with point 2 by Reviewer 2 who suggests that temporally-persistent anti-savings may reflect recent movement and action selection history (use-dependent learning, UDL) during the long washout period prior to relearning. We agree that UDL would indeed slow learning in the VMR training period following the 800 repeated no-rotation reaches to the target in the 800-trial washout condition compared to following a much shorter washout period such as in the 40-trial washout condition. However, it is critical to note that (1) because we are examining savings, the "slower" relearning that we find refers to slower than initial learning and (2) that this initial/baseline learning in our experiments was always preceded by 220 consecutive baseline (no rotation) movements, which would also elicit a UDL effect. The key question regarding the issue that the reviewer raises here is thus not whether a UDL effect would occur following the 800-trial washout period, but whether such an effect would show a size increase between 220 and 800 trials that might explain the anti-savings observed in our data?

First, we note that reported use-dependent effects typically emerge at spans substantially shorter than 220 (or for that matter, 800) trials. Studies examining UDL effects in reaching movements showed effects after as few as 1 [(Verstynen & Sabes, 2011), Exp 3] or 15 trials (Diedrichsen et al., 2010); in the one study that looked at the time course for UDL effects beyond 15 trials, these effects asymptoted by 150 trials [(Verstynen & Sabes, 2011), Fig. 4], which is smaller than the duration of the 220-trial baseline that preceded initial learning in our experiment. This suggests that UDL effects, if they indeed affect VMR training in our study, would do so equally for both initial learning, which was preceded by 220 no-rotation trials, and relearning following long-washout, which was preceded by 800 no-rotation trials. Consequently, UDL effects should have no effect on the difference between these learning curves and thus on the savings we measure after 800 washout trials, and are, therefore, unlikely to explain the temporally-persistent anti-savings we observed.

As a side note, if UDL effects were indeed somewhat lower after 40 trials (the short washout period) compared to 220 trials (the baseline period) as hinted at by Figure 4 in the Verstynen and Sabes study, it would lead to somewhat reduced slowing for relearning after 40 washout trials than for the initial learning. This would suggest that, if anything, we underestimated temporally-persistent anti-savings in the 40-trial washout data rather than overestimating it in the 800-trial washout data as suggested.

However, (Verstynen & Sabes, 2011) did not study UDL training periods as long as 800 trials (540 trials was their maximum), and investigated UDL outside the context of adaptation to a visuomotor rotation. We could not

find other studies examining the time course of UDL effects in reaching movements. Thus, out of caution, we performed an additional experiment to test the prediction that the number of no-rotation reaches before initial learning (220 trials) vs. before the long-washout relearning (800 trials) would lead to similar learning curves in the training period. We, therefore, examined initial learning after a baseline of 800 rather than 220 trials (where 800 trials matches the movement/action selection history of the 800-trial washout before relearning in Experiments 1 and 2). The idea is, as put by reviewer 2, to test "whether the persistent response is [similar or] different following a newly formed adaptation memory".

If the reduction in temporally-persistent (TP) relearning with respect to initial learning we observed were indeed due to the longer 800 trial movement/action selection history as suggested by reviewers 1 and 2, we would expect initial TP learning in this new dataset to match the slowed TP relearning that we previously observed after 800 washout trials rather than the initial TP learning that we previously observed after 220 baseline trials. However, the results from Experiment S1 instead found that the initial TP learning following 800 baseline trials in this new dataset was a closer match to the initial TP learning that was previously observed after 220 baseline trials (see Fig S1), as this dataset did not show the slower initial TP learning that would be predicted by increased UDL following 800 rather than 220 trials. Instead, as shown in Fig S1, the data are in line with the prediction of the V&S data that UDL effects should asymptote well before 220 trials. In line with this prediction, the TP relearning following 800-trial washout trials observed in the previous data was also significantly slowed compared to this new 800-trial baseline data, with significant anti-savings observed when the data from trials 10, 40 & 70 were averaged together ($t(50) = -3.2$, $p = 0.0012$) and also when the data from these timepoints were analyzed separately ($t(50) = -2.2$, $p=0.0159$ at trial 10; $t(50) = -2.5$, $p=0.0082$, at trial 40; $t(50) = -2.2$, $p=0.0155$ at trial 70).

A final, minor point, consistent with the findings presented above, is that a more detailed version of the anti-savings analysis originally presented, provides some evidence for anti-savings in the 40-trial washout data where (as discussed 3 paragraphs above) the presence of UDL effects would promote faster rather than slower relearning compared to baseline (because the 40-trial washout before relearning should induce a somewhat weaker UDL effect than the 220-trial baseline before initial learning). Whereas we originally only looked at TP anti-savings at trial 10 ($t(37) = -1.7$, $p = 0.1073$, 2-tailed paired t-test), a more detailed analysis that also includes the data from trials 40 and 70 shows significant TP anti-savings for the 40-trial washout ($t(39) = -2.6$, $p = 0.0131$, 2-tailed paired t-tests), and when the trial 40 and trial 70 timepoints are analyzed in isolation for the 40-trial washout the results for TP anti-savings are mixed ($t(39)=-2.1$; $p = 0.0417$ at trial 40; $t(38)=-0.7$; $p = 0.5053$ at trial 70, 2-tailed paired t-tests). In comparison, when the analogous analysis for TP anti-saving is performed on the 800-trial washout data the original finding of significant anti-savings at trial 10 ($t(38) = -2.3$, $p=0.0244$, 2-tailed paired t-test) is consistently observed when the data from trials 10, 40 and 70 are averaged ($t(39) = -5.1$, $p = 0.0000098$) and when the trial 40 and trial 70 data are examined in isolation (($t(39)=-3.3$; $p = 0.0020$ at trial 40; $t(38)=-3.1$; $p = 0.0032$ at trial 70, 2-tailed paired t-tests). In summary, the new data from Experiment S1 shows that initial learning following baselines of 220 and 800 trials are quite similar as expected from previous work, and more importantly, that when relearning after the 800-trial washout is compared to the new initial learning data with a 800-trial baseline period, significant TP anti-savings is preserved. Moreover, a more detailed analysis of TP anti-savings in the original data provides some evidence for anti-savings even in the 40-trial washout where UDL effects would lead to underestimation of it and even stronger evidence in the 800-trial data.

Please note that we have now expanded the text in the results section of the manuscript (lines 271-324) to address the points raised above regarding the possible effect of UDL on the data.

1.4 Minor
Line 286-293
Experiment 4 is typed as Experiment 3

Thank you for catching this typo! We have now corrected it.

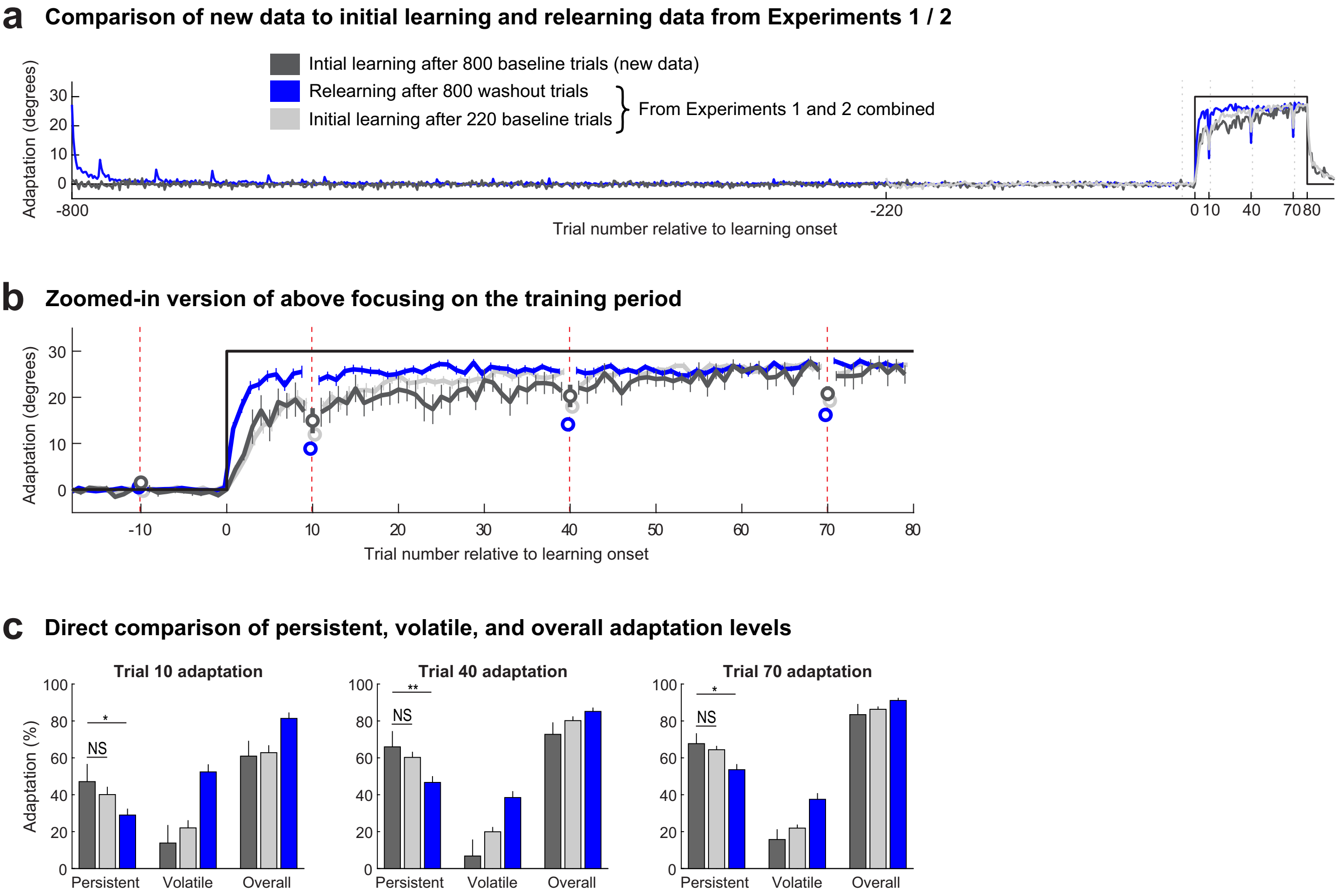

Figure S1: *Anti-savings in temporally-persistent adaptation cannot be explained by prolonged reaching under baseline conditions.* Here we investigated whether the anti-savings found in Experiments 1 / 2 (most pronounced following an 800-trial washout) might be due to the prolonged 800-trial washout period strengthening the baseline, unadapted state to the point that it resists the formation of a temporally-persistent memory of adaptation during relearning. Previous literature suggests that this kind of repetition effect – a form of use-dependent learning (Classen et al., 1998; Diedrichsen et al., 2010) – tends to level off after only 50-150 trials (Verstynen & Sabes, 2011); thus, it should equally affect initial learning (which follows 220 baseline trials) and relearning after 800 washout trials, suggesting no net effect in the anti-savings we observe. However, this use-dependent learning effect has not been studied within the specific context of our task. Thus, in Experiment S1, we examined 12 new participants who adapted to a 30° visuomotor rotation following an 800-trial baseline period, to match the long washout period in Experiments 1 and 2. We found that the prolonged baseline in Experiment S1 (light gray) did not reduce the temporally-persistent component during adaptation compared to the shorter, 220-trial baseline in Experiments 1 and 2 (dark gray); instead, relearning after a 800-trial washout in Experiments 1 and 2 led to significant reductions in temporally-persistent adaptation, as we discuss in the main text. Together, these findings show that anti-savings in temporally-persistent adaptation were not due to the use-dependent learning during the long 800-trial washout period. **(a)** Comparison of average adaptation curves for (i) initial learning after 800 baseline trials from Experiment S1 (light gray), (ii) initial learning after 220 baseline trials from Experiments 1 / 2 (dark gray), and (iii) relearning after 800 washout trials from Experiments 1 / 2 (blue). **(b)** Close-up of the adaptation phase, with temporally-persistent measurements indicated by the empty circles as in Figure 3A. Note the similarity in the adaptation curves for the both initial learning cases (both after 220 and 800 trials of baseline) in contrast to the relearning curve. Errorbars indicate SEM; red lines indicate 60-second delays used to isolate temporally-persistent adaptation. **(c)** Comparison of the levels of overall, temporally-persistent, and temporally-volatile adaptation for these three cases, at trials 10, 40, and 70 after the onset of the visuomotor rotation perturbation. Temporally-persistent adaptation displays no signs of reduction after the 800-trial baseline (new data) relative to the 220-trial one (Exp. 1/2 data); however, it is significantly higher than temporally-persistent adaptation during relearning after the 800-trial washout in Exp. 1/2.

Reviewer #2: The current behavioural study investigates across three visuo-motor adaptation experiments whether slow-and fast decaying adaptive responses to a previous rotation during wash-out is associated with different types of memory: 1) "savings" - the speed of re-adaption to a previously encountered visuo-motor rotation and 2) long-term retention - the presence of a consolidated motor adaptation memory 24h after acquisition. Slow and fast decaying adaption retention during washout and retraining were obtained following 60 sec break or trial-by-trial, respectively. Further explicit verbal instructions were used to manipulate implicit and explicit components of fast and slow decaying responses. The principal finding of this study is that fast decaying adaptive responses were associated with faster relearning (savings), but not with retention on the next day, challenging the assumed notion that savings are a marker of long-term memory formation. In contrast, slower decaying adaptive responses were related to long-term memory retention on the next day, but not with with relearning.

This study is interesting and timely - it questions whether the widely used measure of savings during relearning (obtained on the same day after wash-out) can be seen as a proxy for memory consolidation.

We thank the reviewer for the kind words.

My main concerns relate to the conceptual interpretation of the employed measures and a general lack of integration of the findings with literature outside the motor adaptation community (e.g. contextual interference).

2.1 The 60 sec break manipulation is very elegant and triggers persistent responses from a previous task condition, respectively, across washout and adaptation phases. Yet, it remains conceptually uncertain why it is framed as a component of motor learning and adaptation, rather than as a reflection of the prior movement history (use-dependent learning) and history of action selection (e.g. Valyear and Fitzpatrick 2018) here with respect to the movement direction. For instance, the response following 60 sec breaks during the first adaption period is biased in the reach direction during the baseline (no rotation). It is inverted in the direction of motor commands before any motor learning / adaptation has taken place, which challenges the idea that this is a measure of a newly formed motor memory (persistent or not). Accordingly, after the first visuo-motor rotation period the persistent response reflects bias in the opposite direction, due to the recent induced adaptation response history and then flips again in subsequent adaptations towards the washout response. The fact that the initial and relearning adaptation response after a 60 sec break show equal bias towards the preceding baseline/washed out response could be seen as simply reflecting recent movement and action selection history. Perhaps there is a way to tease out the conceptual difference by testing whether the persistent response is different following a newly formed adaptation memory, whether it is unique to this scenario. As a minimum the manuscript would benefit from clarifying whether and how these components can be disentangled conceptually in the context of this task.

The reviewer here proposes a possible explanation of the temporally-persistent anti-savings finding shown in Figure 6e of our manuscript that matches the explanation suggested above by reviewer 1 (point 1.3): that a large number of baseline/washout movements may generate a bias towards the baseline movement direction which would slow subsequent relearning. We address this possibility in detail above in section **1.3** on pages [4-6] of this letter).

Please see this detailed response including Fig S1 in section **1.3** above, but in summary we agree the suggested use-dependent learning (UDL) effect should be present in the 800-trial washout relearning data, but critically, that it should also be present (and thus controlled for) in the initial learning data that follows a long (220 trial) baseline, in line with evidence suggesting that UDL effects for movement direction asymptote before 150 trials in similar goal-directed reaching movements (Verstynen & Sabes, 2011) . Moreover, we gathered additional data (see Fig S1 above) to double-check whether the size of the UDL effect present after 220 baseline trials is indeed preserved when increasing the number of baseline trials from 220 to 800 as predicted from the available literature, and we find that it is.

2.2 The effect of volatile motor learning being associated with faster adaptation and temporally persistent motor learning (assessed following pauses) being associated with better memory retention on the next day echoes seminal older findings of contextual interference found across different motor (Battig 1972, Shea and Morgan 1979; Cross et al. 2007 and many others), as well as cognitive tasks (de Croock, van Merrienboer, & Paas, 1998). Here for blocked vs spaced-out practise of new skills similarly lead to faster acquisition vs long-term retention, respectively. The manuscript should outline how the current paradigm – in which volatile and persistent components of memory are obtained from the same session and within participants – relates and adds to this seminal literature.

We appreciate the Reviewer's insight in pointing out this potential parallel to our work. We welcome the opportunity to relate our work to the framework of contextual interference, and we have thus expanded the Discussion as suggested to discuss this connection.

Lines 586-622 in the revised manuscript:

*"**The coexistence of temporally-volatile and temporally-persistent memories provides a mechanism for contextual interference:** The possible mapping of temporally-volatile adaptation onto a fast learning / low retention process and of temporally-persistent adaptation onto a slow learning / high retention process provides an intriguing potential explanation for previous work on contextual interference in both motor and cognitive tasks. Contextual interference refers to phenomenon that memories formed in high-interference environments, where the task being performed is randomly switched from one trial to the next, are learned more slowly but show higher retention than memories formed in low-interference environments, where a single task is serially practiced (Battig, 1972; Cross et al., 2007; de Croock et al., 1998; Magill & Hall, 1990; Schneider et al., 2002; Shea & Morgan, 1979). If, as in the VMR adaptation task studied here, both temporally-volatile and temporally-persistent memories contribute to learning in tasks where contextual interference has been observed, then contextual interference effects can be predicted based solely on the temporal spacing inherent in the paradigms that elicit it. The idea here is that the high-interference condition in which tasks are randomly intermingled from one trial to the next would necessarily increase the temporal spacing between the trials within each task compared to the low-interference condition in which tasks are serially practiced. This increase in temporal spacing would allow temporally-volatile memories to decay, at least partially, and thus reduce the amount of temporally-volatile learning, which would slow the overall learning and promote increased temporally-persistent learning. Moreover, the resulting reduction in temporally-volatile learning and increase in temporally-persistent learning would both act to increase the proportion of learning that is temporally-persistent in the high-interference condition, which would, in turn, increase long-term retention according to the current findings. The slowed overall learning and increased retention predicted here for the high-interference condition are, in fact, the defining features of contextual interference. Correspondingly, the converse, faster overall learning but reduced retention, would be predicted for the low-interference condition where serially practiced tasks with reduced temporal spacing would allow temporally-volatile memories to rapidly build during training to improve performance, but would decay before a retention or transfer test, resulting in poor retention. Thus, the coexistence of temporally-volatile and temporally-persistent memories provides an explanation for contextual interference that does not require any interference itself. Further work will be required to determine the fraction of observed contextual interference effects that stem from this mechanism."*

2.3 There is no computational modelling employed in this manuscript, yet a direct link is postulated between volatile/persistent and fast/slow learning process of adaptation, respectively in the Discussion. Unless a computational model used by the authors in their previous work is employed, this claim remains speculative and this putative parallel should be tagged as such in the Discussion.

The Reviewer is correct that the link we suggest between temporally-volatile and temporally-persistent learning and the individual adaptive processes from the two-state model of adaptation is speculative. We certainly did not intend to imply that it was not, and we have now further clarified this.

"These parallels argue, although speculatively so, that the temporally-volatile and temporally-persistent learning from our dissection of adaptation corresponds to the implicit components of fast and slow processes from the two-state model."

2.4 In its current form, the manuscript is written to a specialized "niche" readership of motor adaptation researchers and full of jargon. I suggest to increase clarity by adding an explanatory figure, similar to the one in Fig. 4b to the beginning of the manuscript. For instance, it would be useful to outline the mapping between response measures surrounding 1-minute breaks, derived measures and how they map onto each memory component (e.g. volatile vs persistent) in the three experiments, respectively. This information is currently buried in the Methods section at the end of the manuscript. It should take a much more prominent position to increase understanding and critical evaluation by researchers outside the immediate specialist area from the outset of the manuscript.

We thank the Reviewer for this suggestion. We now have added additional information to diagram the experimental dissection of learning into temporally-volatile and temporally-persistent components (an additional panel for Expts 1-2 in Fig 1, and improving Fig 5, panel a for Expt 4; both panels are reproduced below), as suggested. We have also improved the results-section text (lines 129-136) to clarify how we experimentally dissect initial learning and savings. Finally, we have also improved the corresponding text in the Methods section as well (lines 736-741).

Figure 1d (new panel): explaining the dissection of adaptation into temporally-persistent and temporally-volatile components for Experiments 1/2

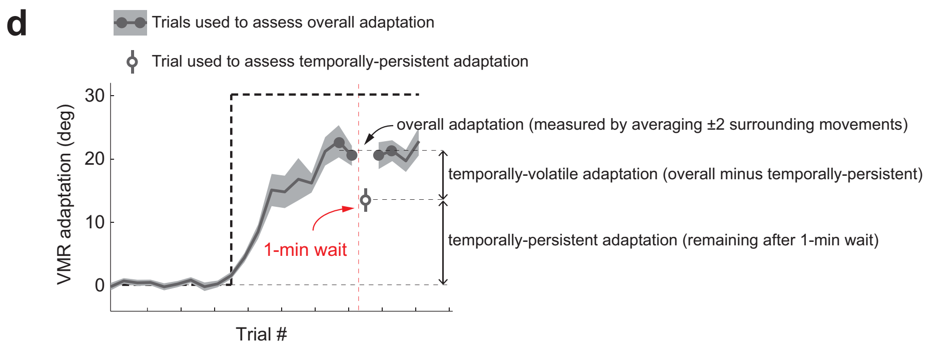

Figure 5a (improved panel): explaining the dissection of adaptation into temporally-persistent (TP) and temporally-volatile (TV) components for Experiment 4 (yellow indicates trials used to measure overall adaptation)

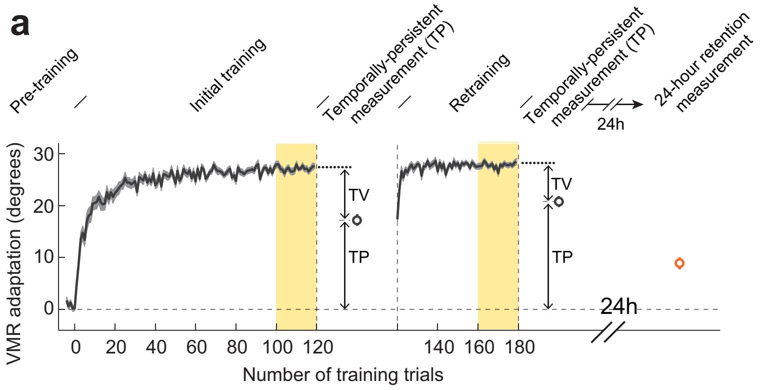

2.5 Minor comments:

* p. 5 line 126: missing p values
* p. 6 line 155 and 171: missing p values

Thank you for catching these errors. For lines 126 & 155, we've now filled in the p-values. For line 171, we ended up revising the surrounding text to simplify the minor point it made and removed quantitative comparisons.

Reviewer #3: In this study Hadjiosif and collaborators aimed at dissecting the learning mechanisms at the basis of savings and long-term memory in visuomotor adaptation. Through four experiments in which they manipulated washout duration, implicit/explicit learning and long-term memory, the authors find a dissociation between the mechanisms supporting savings and long-term memory. Contrary to what is generally believed in the field, they find that savings is explained by a temporally labile rather than a temporally persistent component of adaptation, suggesting that it may reflect meta-learning rather than memory strength. In contrast, they find that long-term memory depends on a temporally persistent component of adaptation. Interestingly, savings appears to arise from implicit, not explicit adaptation.

The study is novel and certainly relevant to the field. It sheds light on several key aspects of motor learning using a simple experimental design via clean experimental manipulations and straightforward analyses. The reported dynamics on the decay of the temporally stable adaptation process, and its modulation by the amount of training are both critical findings. Until now, complete washout was estimated based on the overall level of performance. Tracking persistent adaptation using clever 1 min intervals interspersed through the washout period unveiled the true dynamics of memory decay, which are much lower than previously thought. Another important contribution of the study is the finding that savings is driven by the temporally labile component of learning. This is both interesting and, again, unexpected since this phenomenon has been naturally associated with the amount of training and the strength of memory both in the procedural and declarative fields. Finally, and in contrast to previous work, it shows that savings arises from the implicit learning process. Overall, the methodology and statistical analyses are sound, and easy to follow and the figures are clear, although some captions need improvement.

We thank the reviewer for the kind words.

Yet, at times the manuscript is hard to follow. Below, I list some suggestions and raise a few concerns that, if addressed, will help improve the quality of the work.

3.1 The abstract is somewhat disorganized. The aim and hypothesis are there, but some key information is missing, for example the definition of the temporally volatile and persistent components of learning, which are key processes quantified here. It is stated that adaptation was experimentally dissected based on short-term (1-minute) temporal persistence. Yet, this sentence is not sufficient to understand what is being measured in terms of the overall aim of the study. Also, note that some sentences, like the results sentence, are long and intricate. If possible, reword for better comprehension.

We thank the Reviewer for pointing out several ways in which our abstract can be better organized, which we believe resulted in a text that's more clear and friendly to the reader. We have thus edited our abstract as shown below (main changes in red):

"Memories are easier to reacquire than learn from scratch. This advantage, known as savings, has been widely assumed to result from the reemergence of stable long-term memories. In fact, the presence of savings has often been used as a marker for whether a memory had been consolidated. However, recent findings have demonstrated that motor learning rates can be systematically controlled, providing a mechanistic alternative to the reemergence of a stable long-term memory. Moreover, recent work has reported conflicting results about whether implicit contributions to savings in motor learning are present, absent, or inverted, suggesting a limited understanding of the underlying mechanisms. To elucidate these mechanisms, we investigate the relationship between savings and long-term memory by experimentally dissecting the underlying memories based on short-term (60-second) temporal persistence. Components of motor memory that are temporally-persistent at 60 seconds might go on to contribute to stable, consolidated long-term memory, whereas temporally-volatile components that have already decayed away by 60 seconds cannot. Surprisingly, we find that temporally-volatile implicit learning leads to savings whereas temporally-persistent learning does not, but that temporally-persistent learning leads to long-term memory at 24 hours whereas temporally-volatile learning does not. This double dissociation between the mechanisms for savings and long-term memory formation challenges widespread assumptions about the connection between savings and memory consolidation. Moreover, we find that temporally-persistent implicit learning not only fails to contribute to savings, but that it produces an opposite, anti-savings effect, and that the balance between this temporally-persistent anti-savings and temporally-volatile savings provides an explanation for several

seemingly conflicting recent reports about whether implicit contributions to savings are present, absent, or inverted. Finally, the learning curves we observed for the acquisition of temporally-volatile and temporally-persistent implicit memories demonstrate the coexistence of implicit memories with distinct time courses, challenging the assertion that models of context-based learning and estimation should supplant models of adaptive processes with different learning rates. Together, these findings provide new insight into the mechanisms for savings and long-term memory formation."

3.2

Section 3.2a. Overall, the Introduction is well written but does not emphasize the main aim enough, i.e. dissecting the learning mechanisms at the basis of savings and long-term memory.

Thanks for pointing out this oversight. We agree with the reviewer that the dissection of learning into volatile vs. persistent components needs more emphasis. We have now flushed out how the potential consequences of savings stemming from these components would shed light on the connection between savings and memory consolidation.

"Since it decays away in just 60 seconds, temporally-volatile adaptation would not lead to long-term memory which is associated with stability across timescales that are orders of magnitude greater(Joiner & Smith, 2008; Landi et al., 2011; Shadmehr & Brashers-Krug, 1997). Hence, mechanisms leading to long-term memory would be contained within the temporally-persistent component. Thus, consolidation-dependent savings would predict faster learning solely for temporally-persistent adaptation. Conversely, if we found faster relearning solely for temporally-volatile adaptation, that would indicate a savings mechanism that is not driven by long-term memory."

Section 3.2b. In fact, the literature background is focused on savings solely (there is in fact too much detail, some of which may be more appropriate in the Discussion). Given that the double dissociation between savings and long-term memory is a major contribution of the study, both the literature on savings and long-term memory should be considered in the Intro.

As suggested, the revised manuscript now provides a more meaningful introduction to key information about long-term memory and how it may relate to savings. See below:

"Previous research has generally maintained that savings results from the recall of a previously consolidated long-term memory. The ability to form enduring long-term memories is one of the most remarkable biological abilities, with some memories lasting a lifetime despite incessant neural plasticity. Elderly Danes recalled a host of mundane details – such as the weather – surrounding the news of their country's invasion and liberation during WWII more than 50 years later (Berntsen & Thomsen, 2005), and similar examples of memories surrounding shocking or consequential events abound(Brown & Kulik, 1977; Conway et al., 1994; Hirst et al., 2009). People recognize high school classmates decades later (Bahrick et al., 1975), tacitly use synthetic grammar rules years after training (Allen & Reber, 1980), and identify specific images months (Mandler & Ritchey, 1977) or even years (Larzabal et al., 2018) later. Long-term memories emerge through consolidation, a process driven by molecular mechanisms that facilitate synaptic activity (Kandel, 2001; Katche et al., 2013) and can be mediated through the hippocampus (Riedel et al., 1999; Squire et al., 2015). In motor tasks, the presence or absence of savings itself has often been taken as a litmus test for whether a previously trained memory has been consolidated, over timescales ranging from hours to months(Caithness et al., 2004; Krakauer & Shadmehr, 2006; Landi et al., 2011). Consequently, in terms of underlying mechanisms, savings has been further suggested to result from (1) the unmasking of a slower-learning, strong-retention process in a multi-rate learning model(Smith et al., 2006); (2) context- or relevance- based switching between such multiple slow processes, each specific to a different memory(Berniker & Kording, 2011; Heald et al., 2021; Lee & Schweighofer, 2009; Pekny et al., 2011); or (3) reverting to the memory of a previously learned motor plan that was reinforced by success or mere repetition(Huang et al., 2011; Shmuelof et al., 2012). All of these propositions focus on savings as the manifestation of a latent, stable, consolidated motor memory that is robust to both interference and the passage of time."

Section 3.3c. Moreover, given the central relevance of the temporally volatile and persistent learning processes to the aim of the study, they should be defined in this section, and linked to the slow and fast modules. In the current version of the manuscript these processes are defined in terms of the experimental manipulation (persist

vs doesn't persist the 1 min interval) not in terms of their function. This conceptual information is important to put the aim of the study in context.

We again agree with the reviewer here. Please see section 3.2a above where this is addressed.

3.3 The description of how volatile adaptation was quantified (lines 598-602 of the Methods) is cumbersome. Please provide a clearer explanation since it is key to understand the outcomes of the study.

Thank you for pointing out this issue. Based on a related comment from reviewer 2, we have now clarified the explanations of how volatile and persistent adaptation were quantified in both the Methods and Results sections, and we have, in addition, added a panel to Fig 1 (see Fig 1d) and additional information to Fig 5 (see Fig 5a) to further improve the clarity of these explanations. Please see section 2.4 above for details.

3.4 Figure 1C shows that performance during adaptation doesn't seem to quite reach an asymptote in Experiment 1, which seems to be the case in Experiment 2. This may impact on the subsequent estimation of savings since there may still be room for improvement. How is this taken into account in the analyses? Data normalization won't help. Please comment on this potential limitation, and provide a quantification of the achieved asymptotic performance for these experiments.

When we look at this quantitatively, we don't see a big difference in the extent to which the learning curves from Expts 1 and 2 reach asymptote. The extent to which asymptote is achieved is usually assessed by the strength of the trend in the later part of a time series data set. However, when we compare the last 10 of the 80 trials in the VMR block (excluding the post-delay trial) to the 10 trials before that, we find increases that are only a fraction of a degree for both Expts 1 and 2 (specifically, the differences between these blocks were 0.2° and 0.3° overall for Expts 1 and 2, respectively). And when we break these the overall differences down by looking at the three different learning curves in each of these experiments, none of the three show either large differences in performance between these two consecutive late blocks or between experiments:

For the difference between the performance level on the last 10 trials in the VMR block (not including post-break trials) and the previous 10:

Initial learning: 0.4±0.6° vs 0.0±0.4° for expts 1 vs 2, p = 0.5768
Learning after 40 washout trials: -0.1±0.4° vs. -0.1±0.5°, p = 0.9600
Learning after 800 washout trials: 0.3±0.6° vs. 1.1±0.4°, p = 0.2662

FYI, this corresponds to the following performance levels in the two late blocks:

For the last 10 trials (not including post-break trials):
Initial learning: 25.9±0.6° vs. 27.3±0.6° for expts 1 vs 2
Learning after 40 washout trials: 26.9±0.6° vs. 27.5±0.4°
Learning after 800 washout trials: 25.8±0.7° vs.28.3±0.4°

For the previous10 trials:
Initial learning: 25.5±0.7° vs. 27.3±0.5° for expts 1 vs 2
Learning after 40 washout trials: 27.0±0.5° vs. 27.6±0.5°
Learning after 800 washout trials: 25.5±0.9° vs. 27.1±0.4°

Moreover, essentially identical results are obtained when comparing *the last 10 trials in the VMR block and the 20 (rather than 10) trials before that.*

Initial learning: 0.9±0.7° vs. 0.0±0.2° for expts 1 vs 2, p = 0.2534,
Learning after 40 washout trials: -0.1±0.3° vs -0.3±0.5°, p = 0.6973
Learning after 800 washout trials: 0.8±0.4° vs 1.5±0.3°, p = 0.1959

Maybe we're misunderstanding here, but we don't see an effect that should be accounted for in the analyses. It is certainly true that, as the reviewer alludes to, the difference between the performance level at a certain point in the initial learning curve and the asymptotic level would in principle put a limit on how much savings could occur at this point in the learning curve. And certainly, this limit would be larger earlier on in the learning curve and smaller late in learning when the performance in nearly asymptotic. It is likely for this reason that we (as well as other studies) find the greatest levels of savings fairly early in the learning curve. In fact, in hindsight, we would likely have obtained even clearer data had we first dissected adaptation into TV and TP components at trial 4 or 5 rather than trial 10. But, in summary, after examining it quantitatively, we find little difference between performance levels in consecutive blocks late in the VMR period suggesting that the data are near asymptote, and little difference between these consecutive-block differences when comparing experiments 1 and 2.

3.5 *I understand that Experiment 4 aims at examining whether the temporally persistent and labile components of learning relate to long-term memory. Yet, what is the rationale for including two sessions of practice and having two measurements of the temporally persistent process? How is that an advantage when trying to link this process to retention, considering there would be cumulative effects of further training? Which measure of persistent adaptation was correlated to memory retention, the one corresponding to the first session, the second session, or their average?*

We used the average of the two measurements in the Expt 4 regression analysis presented in Fig 6. Unfortunately, our original submission wasn't as clear as it could have been about this, and it also omitted the rationale for this design decision. We designed Expt 4 to make two measurements of temporally-persistent learning for each participant simply because we thought that averaging two measurements might reduce the effects of measurement noise. Thus we expected that both TP measurements would predict 24 hour retention, but that the average might make a cleaner prediction. Note that the initial training period was 120 trials in duration, enough that the performance would asymptote so that cumulative effects of further training should be small, which appears to be borne out in the learning curve shown in Fig 5a. We have now made changes to both our methods and results sections to provide this information.

Materials and methods (lines 753-757 in the revised version)):

"...temporally-persistent adaptation was measured as the average of six reaches (three in each of the two no-feedback testing blocks) that were towards the training target. We designed Experiment 4 with two test blocks because we thought that averaging the data from both blocks might reduce the effects of measurement noise, as we expected that both temporally-persistent measurements would predict 24 hour retention, but that the average might make a cleaner prediction"

Results (lines 359-365):

*"After this initial training, participants were tested for the temporally-persistent adaptation present after a rest break (average break duration: 124±9s, which would allow >99% of temporally-volatile adaptation to decay based on a time constant of 15-25sec). The above measurements were then repeated, with participants retrained for 60 trials, and retested for temporally-persistent adaptation (the average of these two measurement sessions was used to quantify temporally-persistent adaptation for each individual). Participants then returned the following day to be tested for retention (**Fig. 5a**, see Materials and Methods)."*

Were any of them more strongly correlated to long-term memory? I can't find this information in the Methods.

Based on the reviewer's questions here, we repeated the Expt 4 regression analysis using individual test block/session measurements of TP and TV learning, i.e. from the 1st or 2nd sessions separately. We find that these individual-session measurements show remarkably similar results to one another and to the averaged data that was analyzed in the paper, displaying significant regression coefficients for TP but not for TV in all cases.

1st session data: Partial R^2 for TP = 43.1%, p = 0.000498, partial R^2 for TV = 1.0%, p = 0.643.
2nd session data: Partial R^2 for TP = 53.0%, p = 0.0000547, partial R^2 for TV = 8.6%, p = 0.163.

Averaged data: Partial R^2 for TP = 51.7%, p = 0.0000748, partial R^2 for TV = 3.8%, p = 0.363.

We have added this information in the revised manuscript. A minor point is that the partial R^2 value for TP is nominally higher for the 2nd session than the 1st as might be expected (0.530 vs 0.431), as it is closer to the 24-hour retention measurement - but this is only a modest effect.

3.6 I don't see how Figure 5 provides evidence supporting that Temporally-persistent adaptation leads to long-term memory, which is claimed in the figure's title. This seems rather more related to Figure 6, so please modify the title.

Thanks for catching this! This errant title was an unfortunate holdover from a previous version of this Figure that had combined what are now Figs 5 & 6. We have now changed the Fig 5 title to "Measuring temporally-volatile and temporally-persistent components of adaptation and subsequent long-term retention".

Also, given that the results depicted in Figure 6 are not causal but correlational it would be more appropriate to refer to it as an association instead of assuming causality. The latter applies for the rest of the manuscript.

We agree with the Reviewer that the results in Figure 6cd are better described as an association. We have now improved the relevant text to convey this point. In particular, in the last paragraph of our Results section, we replaced mentions of "leads to…" or "explains" with "is associated with" (lines 411, 413, 432, and caption of Figure 6a).

In addition, the caption of figure 5 is somewhat confusing. What is being measured and represented here as temporally stable measurements? Is it the temporally persistent adaptation component?

Thanks for catching this error – we have now corrected it. Yes, we meant to say "temporally-persistent" rather than "temporally-stable". Sorry for the typo.

And how is it measured compared to Figure 1? Although stated somewhere in the Results section, neither the Methodology nor the figure caption indicate that temporally persistent adaptation was quantified 1-minute post training. Please provide all the necessary information in the figure caption, and use the same terminology to refer to the variables of interest consistently across figures, e.g., persistent adaptation instead of temporally stable measurement, etc.

In Experiment 4, TP adaptation was also quantified after a time delay in the form of a block break. As we also discuss in our response to reviewer 1 (point 1.2), the average break duration was 124±9s. Given a time constant for temporal decay of τ = 15-25s (Hadjiosif et al., 2012; Sing et al., 2009; Zhou et al., 2017), this would amount to ~6τ, letting >99% of temporally-volatile adaptation decay, isolating temporally-persistent adaptation; this would be very similar to the effect of the 60s (~3τ) breaks employed in Figure 1, which would let ~95% of temporally-volatile adaptation decay. We now provide this information in the Figure 5 and the Methods section (lines 751-753), rather than just the Results.

3.7 The conclusions drawn from this study may depend on the particular experimental paradigm/design chosen here. The authors claim that their task elicited more implicit than explicit learning. Would the results hold if the instructions were changed so that the task elicited relatively more explicit or equal amounts of explicit and implicit learning? In other terms, how generalizable are these findings to other paradigms/designs of VMA? This is a relevant issue and should be addressed in the Discussion.

We, of course, agree both that conclusions drawn from this study (or any) may depend on the particular experimental paradigm/design and that a key feature of the current experimental design is that elicited more implicit than explicit learning, as we discuss in both the results (lines 348-353) and discussion sections (lines 553-560). This made the contributions to implicit savings that we sought to examine considerably easier to discern. Interestingly, previous work provides some evidence that explicit savings is driven by temporally-persistent memory, as explicit savings is clearly observable in multi-target paradigms that would minimize temporally-volatile memory (Avraham et al., 2021; Morehead et al., 2015). And it is clear that paradigm variations

that employ larger VMRs, or with endpoint-only feedback, or where aiming markers are explicitly presented (Bond & Taylor, 2015; Morehead et al., 2015; Taylor et al., 2014) would lead to greater explicit learning. Thus in such paradigms that elicit primarily explicit learning, we might expect primarily TP savings. And for experimental paradigms that elicit similar amounts of implicit and explicit learning, we would expect a smaller dichotomy between TP and TV contributions to savings. However, because the current study did not examine these cases, we cannot know whether interactions between implicit and explicit learning might lead to these expected results or to different results that cannot be predicted from the current findings.

We have expanded our Discussion to address this point (lines 561-571 of the updated manuscript):

"Taken together, the evidence now suggests that both implicit and explicit adaptation can show savings, albeit via different mechanisms. The current study shows that implicit savings is specifically driven by the faster relearning of a temporally-volatile memory, whereas previous work provides evidence that explicit savings is driven by temporally-persistent memory, as explicit savings is clearly observable in multi-target paradigms that would minimize temporally-volatile memory (Avraham et al., 2021; Morehead et al., 2015). As a consequence, for experimental paradigms that primarily elicit explicit learning we would expect savings to primarily be driven by temporally-persistent adaptation, and for experimental paradigms that elicit a balance of implicit and explicit learning we would expect the dichotomy between temporally-persistent and temporally-volatile contributions to savings to be blurred. However, because we did not examine these cases, we cannot know whether our expectations will be borne out vs whether complex interactions between implicit and explicit learning might lead to different results that cannot be predicted from the current findings."

3.8 Minor:

In line 212 there is a disconnected word: "Individually".

Thank you for catching this error! We've fixed it.

Response letter citations

Allen, R., & Reber, A. S. (1980). Very long term memory for tacit knowledge. *Cognition*, *8*(2), 175–185.

Avraham, G., Morehead, J. R., Kim, H. E., & Ivry, R. B. (2021). Reexposure to a sensorimotor perturbation produces opposite effects on explicit and implicit learning processes. *PLoS Biology*, *19*(3), Article 3.

Bahrick, H. P., Bahrick, P. O., & Wittlinger, R. P. (1975). Fifty years of memory for names and faces: A cross-sectional approach. *Journal of Experimental Psychology: General*, *104*(1), 54.

Battig, W. (1972). Intratask interference as a source of facilitation in transfer and retention. *Topics in Learning and Performance*, 131–159.

Berniker, M., & Kording, K. P. (2011). Estimating the relevance of world disturbances to explain savings, interference and long-term motor adaptation effects. *PLoS Computational Biology*, *7*(10), e1002210.

Berntsen, D., & Thomsen, D. K. (2005). Personal memories for remote historical events: Accuracy and clarity of flashbulb memories related to World War II. *Journal of Experimental Psychology: General*, *134*(2), 242.

Bond, K. M., & Taylor, J. A. (2015). Flexible explicit but rigid implicit learning in a visuomotor adaptation task. *Journal of Neurophysiology*, *113*(10), Article 10.

Brown, R., & Kulik, J. (1977). Flashbulb memories. *Cognition*, *5*(1), 73–99.

Caithness, G., Osu, R., Bays, P., Chase, H., Klassen, J., Kawato, M., Wolpert, D. M., & Flanagan, J. R. (2004). Failure to Consolidate the Consolidation Theory of Learning for Sensorimotor Adaptation Tasks. *Journal of Neuroscience*, *24*(40), Article 40. https://doi.org/10.1523/JNEUROSCI.2214-04.2004

Classen, J., Liepert, J., Wise, S. P., Hallett, M., & Cohen, L. G. (1998). Rapid plasticity of human cortical movement representation induced by practice. *Journal of Neurophysiology*, *79*(2), 1117–1123.

Conway, M. A., Anderson, S. J., Larsen, S. F., Donnelly, C. M., McDaniel, M. A., McClelland, A. G., Rawles, R. E., & Logie, R. H. (1994). The formation of flashbulb memories. *Memory & Cognition*, *22*(3), 326–343.

Cross, E. S., Schmitt, P. J., & Grafton, S. T. (2007). Neural substrates of contextual interference during motor learning support a model of active preparation. *Journal of Cognitive Neuroscience*, *19*(11), 1854–1871.

de Croock, M. B. M., van Merriënboer, J. J. G., & Paas, F. G. W. C. (1998). High versus low contextual interference in simulation-based training of troubleshooting skills: Effects on transfer performance and invested mental effort. *Computers in Human Behavior*, *14*(2), 249–267. https://doi.org/10.1016/S0747-5632(98)00005-3

Diedrichsen, J., White, O., Newman, D., & Lally, N. (2010). Use-dependent and error-based learning of motor behaviors. *Journal of Neuroscience*, *30*(15), Article 15.

Hadjiosif, A. M., Petreska, B., & Smith, M. A. (2012). Mechanisms underlying the temporal stability of visuomotor adaptation. *Program No. 679.11. 2012 Neuroscience Meeting Planner. New Orleans, LA: Society for Neuroscience, 2012. Online.* 42nd Society for Neuroscience Annual Meeting.

Heald, J. B., Lengyel, M., & Wolpert, D. M. (2021). Contextual inference underlies the learning of sensorimotor repertoires. *Nature*, *600*(7889), 489–493.

Hirst, W., Phelps, E. A., Buckner, R. L., Budson, A. E., Cuc, A., Gabrieli, J. D., Johnson, M. K., Lustig, C., Lyle, K. B., & Mather, M. (2009). Long-term memory for the terrorist attack of September 11: Flashbulb memories, event memories, and the factors that influence their retention. *Journal of Experimental Psychology: General*, *138*(2), 161.

Huang, V. S., Haith, A., Mazzoni, P., & Krakauer, J. W. (2011). Rethinking motor learning and savings in adaptation paradigms: Model-free memory for successful actions combines with internal models. *Neuron*, *70*(4), Article 4.

Joiner, W. M., & Smith, M. A. (2008). Long-term retention explained by a model of short-term learning in the adaptive control of reaching. *Journal of Neurophysiology*, *100*(5), Article 5.

Kandel, E. R. (2001). The molecular biology of memory storage: A dialogue between genes and synapses. *Science*, *294*(5544), 1030–1038.

Katche, C., Cammarota, M., & Medina, J. H. (2013). Molecular signatures and mechanisms of long-lasting memory consolidation and storage. *Neurobiology of Learning and Memory*, *106*, 40–47.

Krakauer, J. W., & Shadmehr, R. (2006). Consolidation of motor memory. *Trends in Neurosciences*, *29*(1), Article 1.

Landi, S. M., Baguear, F., & Della-Maggiore, V. (2011). One week of motor adaptation induces structural changes in primary motor cortex that predict long-term memory one year later. *Journal of Neuroscience*, *31*(33), 11808–11813.

Larzabal, C., Tramoni, E., Muratot, S., Thorpe, S. J., & Barbeau, E. J. (2018). Extremely long-term memory and familiarity after 12 years. *Cognition*, *170*, 254–262.

Lee, J.-Y., & Schweighofer, N. (2009). Dual Adaptation Supports a Parallel Architecture of Motor Memory. *Journal of Neuroscience*, *29*(33), Article 33. https://doi.org/10.1523/JNEUROSCI.1294-09.2009

Magill, R. A., & Hall, K. G. (1990). A review of the contextual interference effect in motor skill acquisition. *Human Movement Science*, *9*(3–5), 241–289.

Mandler, J. M., & Ritchey, G. H. (1977). Long-term memory for pictures. *Journal of Experimental Psychology: Human Learning and Memory*, *3*(4), 386.

Morehead, J. R., Qasim, S. E., Crossley, M. J., & Ivry, R. (2015). Savings upon re-aiming in visuomotor adaptation. *Journal of Neuroscience*, *35*(42), Article 42.

Pekny, S. E., Criscimagna-Hemminger, S. E., & Shadmehr, R. (2011). Protection and expression of human motor memories. *Journal of Neuroscience*, *31*(39), Article 39.

Riedel, G., Micheau, J., Lam, A., Roloff, E., Martin, S. J., Bridge, H., Hoz, L. de, Poeschel, B., McCulloch, J., & Morris, R. G. (1999). Reversible neural inactivation reveals hippocampal participation in several memory processes. *Nature Neuroscience*, *2*(10), 898–905.

Schneider, V. I., Healy, A. F., & Bourne Jr, L. E. (2002). What is learned under difficult conditions is hard to forget: Contextual interference effects in foreign vocabulary acquisition, retention, and transfer. *Journal of Memory and Language*, *46*(2), 419–440.

Shadmehr, R., & Brashers-Krug, T. (1997). Functional stages in the formation of human long-term motor memory. *Journal of Neuroscience*, *17*(1), 409–419.

Shea, J. B., & Morgan, R. L. (1979). Contextual interference effects on the acquisition, retention, and transfer of a motor skill. *Journal of Experimental Psychology: Human Learning and Memory*, *5*(2), 179.

Shmuelof, L., Huang, V. S., Haith, A. M., Delnicki, R. J., Mazzoni, P., & Krakauer, J. W. (2012). Overcoming motor “forgetting” through reinforcement of learned actions. *Journal of Neuroscience*, *32*(42), Article 42.

Sing, G., Najafi, B., Adewuyi, A., & Smith, M. (2009). A mechanism for the spacing effect: Competitive inhibition between adaptive processes explains the increase in motor skill retention associated with prolonged inter-trial spacing. *Advances in Computational Motor Control. Chicago, IL*.

Smith, M. A., Ghazizadeh, A., & Shadmehr, R. (2006). Interacting adaptive processes with different timescales underlie short-term motor learning. *PLoS Biology*, *4*(6), Article 6.

Squire, L. R., Genzel, L., Wixted, J. T., & Morris, R. G. (2015). Memory consolidation. *Cold Spring Harbor Perspectives in Biology*, *7*(8), a021766.

Taylor, J. A., Krakauer, J. W., & Ivry, R. B. (2014). Explicit and implicit contributions to learning in a sensorimotor adaptation task. *Journal of Neuroscience*, *34*(8), Article 8.

Verstynen, T., & Sabes, P. N. (2011). How Each Movement Changes the Next: An Experimental and Theoretical Study of Fast Adaptive Priors in Reaching. *Journal of Neuroscience*, *31*(27), Article 27. https://doi.org/10.1523/JNEUROSCI.6525-10.2011

Zhou, W., Fitzgerald, J., Colucci-Chang, K., Murthy, K. G., & Joiner, W. M. (2017). The temporal stability of visuomotor adaptation generalization. *Journal of Neurophysiology*, *118*(4), Article 4.
